# Perinatal and child and adolescent mental health: steps towards integration and inclusion from around the world

**DOI:** 10.1192/bji.2025.12

**Published:** 2025-05

**Authors:** Marinos Kyriakopoulos

**Affiliations:** Assistant Professor, Child and Adolescent Psychiatry, School of Medicine, National and Kapodistrian University of Athens, Athens, Greece

Early intervention is increasingly gaining ground as an integral component of mental health policy and practice across the globe. Primary prevention, early detection and effective timely input have been considered a promising approach in reducing psychiatric morbidity and the overall burden of mental health and neurodevelopmental difficulties, with wider societal benefits. For this to succeed, integration of health services with mental health services is paramount, while cultural and educational aspects that may be barriers to inclusion need to be adequately addressed.

In this issue of *BJPsych International*, we have three articles related to perinatal and child and adolescent mental health with implications for effective early intervention. Hameed and Rahman^1^ synthesise the evidence on the burden and impact of common perinatal mental disorders in low- and middle-income countries, also referring to the risk factors and consequences of these conditions, and present the context, policies and some of the innovative work that has been done in Pakistan to address such difficulties. They make specific recommendations for further research in line with the World Health Organization’s stepped-care approach, training across sectors and promoting better integration of perinatal mental health with maternal and child health services. Improved perinatal mental health is likely not only to benefit women during the perinatal period, but may also have significant implications for their infants’ health and development. In the second article, Miller and James^2^ present the child and adolescent mental health services in Aotearoa, New Zealand, serving a multicultural population of about 5 million people. They discuss the cultural context in which the service is provided, as well as the challenges in training, workforce, governance, service provision and funding. Unfortunately, similar challenges are not uncommon in most countries. The authors also highlight significant areas of strength, including cultural sensitivity in service delivery and world-leading research in the field of child development. Finally, Mitroulaki and colleagues,^3^ in their original research paper, present the validation in a large Greek sample of teachers and pedagogical university students of two adapted scales for use in children with autism spectrum disorder (ASD). One of these scales assesses educators’ attitudes and the other measures their sense of self-efficacy towards students with ASD. As we are moving towards an inclusive education and integration of children with neurodevelopmental needs in mainstream educational settings, an accurate evaluation of educators’ attitudes that may hinder successful inclusion and their confidence in performing various teaching tasks effectively with students with ASD is crucial. In this way, appropriate interventions and training may be planned through educational policy to promote large-scale change likely to benefit children with ASD during their early formative years.

The above articles, among many others in the current issue, evidence the vision in different countries to facilitate culturally sensitive, timely and inclusive approaches addressing mental health and neurodevelopmental issues. I hope you enjoy reading it!
